# Perspective in the Mechanisms for Repairing Sperm DNA Damage

**DOI:** 10.1007/s43032-024-01714-5

**Published:** 2024-09-27

**Authors:** Nihong Li, Hong Wang, Siying zou, Xujun Yu, Junjun Li

**Affiliations:** 1 Chengdu Fifth People’s Hospital, The Fifth People’s Hospital of Chengdu University of Traditional Chinese Medicine, Chengdu, 611130 China; 2 College of Medicine and Life Sciences, Chengdu University of Traditional Chinese Medicine, Chengdu, 611137 China

**Keywords:** Spermatozoa, DNA Damage, DNA Repair, Spermatogenesis, Oocyte

## Abstract

DNA damage in spermatozoa is a major cause of male infertility. It is also associated with adverse reproductive outcomes (including reduced fertilization rates, embryo quality and pregnancy rates, and higher rates of spontaneous miscarriage). The damage to sperm DNA occurs during the production and maturation of spermatozoa, as well as during their transit through the male reproductive tract. DNA damage repair typically occurs during spermatogenesis, oocytes after fertilization, and early embryonic development stages. The known mechanisms of sperm DNA repair mainly include nucleotide excision repair (NER), base excision repair (BER), mismatch repair (MMR), and double-strand break repair (DSBR). The most severe type of sperm DNA damage is double-strand break, and it will be repaired by DSBR, including homologous recombination (HR), classical non-homologous end joining (cNHEJ), alternative end joining (aEJ), and single-strand annealing (SSA). However, the precise mechanisms of DNA repair in spermatozoa remain incompletely understood. DNA repair-associated proteins are of great value in the repair of sperm DNA. Several repair-related proteins have been identified as playing critical roles in condensing chromatin, regulating transcription, repairing DNA damage, and regulating the cell cycle. It is noteworthy that XRCC4-like factor (XLF) and paralog of XRCC4 and XLF (PAXX) -mediated dimerization promote the processing of populated ends for cNHEJ repair, which suggests that XLF and PAXX have potential value in the mechanism of sperm DNA repair. This review summarizes the classic and potential repair mechanisms of sperm DNA damage, aiming to provide a perspective for further research on DNA damage repair mechanisms.

## Introduction

Infertility is characterized as the inability of a couple within childbearing age to conceive after 12 consecutive months of regular, unprotected, and well-timed sexual intercourse [1]. The condition is a prevalent health issue, with an estimated 15% of couples experiencing infertility. Surprisingly, male factors contribute to approximately 50% of these cases [2–4]. One major cause of men’s infertility is sperm DNA, connected to abnormal sperm concentration, forward motility, normal morphology, sperm motility, and sperm maturity. Damaged sperm significantly affects embryonic development, potentially resulting in slow or arresting embryonic development, increased miscarriages, and increased rates of disease in offspring [5–7].

The types of sperm DNA damage include base mismatches, base losses, base modifications, DNA adducts and cross-links, pyrimidine dimers, single-strand breaks (SSB), and double-strand breaks (DSB) [8]. DSB is a serious type of DNA damage that can result in chromosome breaks, loss of chromosomal structure, translocations, or genetic recombination. It substantially impacts reproductive outcomes, such as implantation, miscarriage, pregnancy, and live birth rates, as opposed to SSB [9–11]. Intrinsic and extrinsic factors may cause damage to sperm DNA. Intrinsic factors include oxidative stress, damage to spermatogenesis, and apoptosis. Extrinsic factors include environmental, lifestyle, medical, and pathological [12–18]. A commonly used clinical indicator for assessing sperm DNA damage is the DNA fragmentation index (DFI) [19].

Sperm DNA damage is generally repairable, either during spermatogenesis using a variety of repair pathways, or during oocyte and embryo development using mRNAs and proteins [20–22]. During spermatogenesis, several DNA repair pathways are activated to maintain genetic integrity. However, as spermatogenesis nears completion, nuclear gene expression gradually shuts down, resulting in mature spermatozoa becoming silent cells that lack DNA repair capacity [23]. Once fertilized, the primary responsibility for DNA repair shifts to the oocyte. The repair’s extent depends on the oocyte’s quality and the degree and type of sperm DNA damage. High-quality oocytes are more likely to successfully repair damage, particularly if the damage is minor [24–26]. If the egg cell is unsuccessful in adequately repairing DNA damage, it can lead to de novo mutations with profound effects on embryo development [27].

This review will concentrate on the elucidation of the damage to sperm DNA that is caused by internal and external factors, sperm DNA damage repair (DDR) mechanisms, and associated proteins.

## Mechanisms of Sperm DNA Damage

**Fig. 1:**
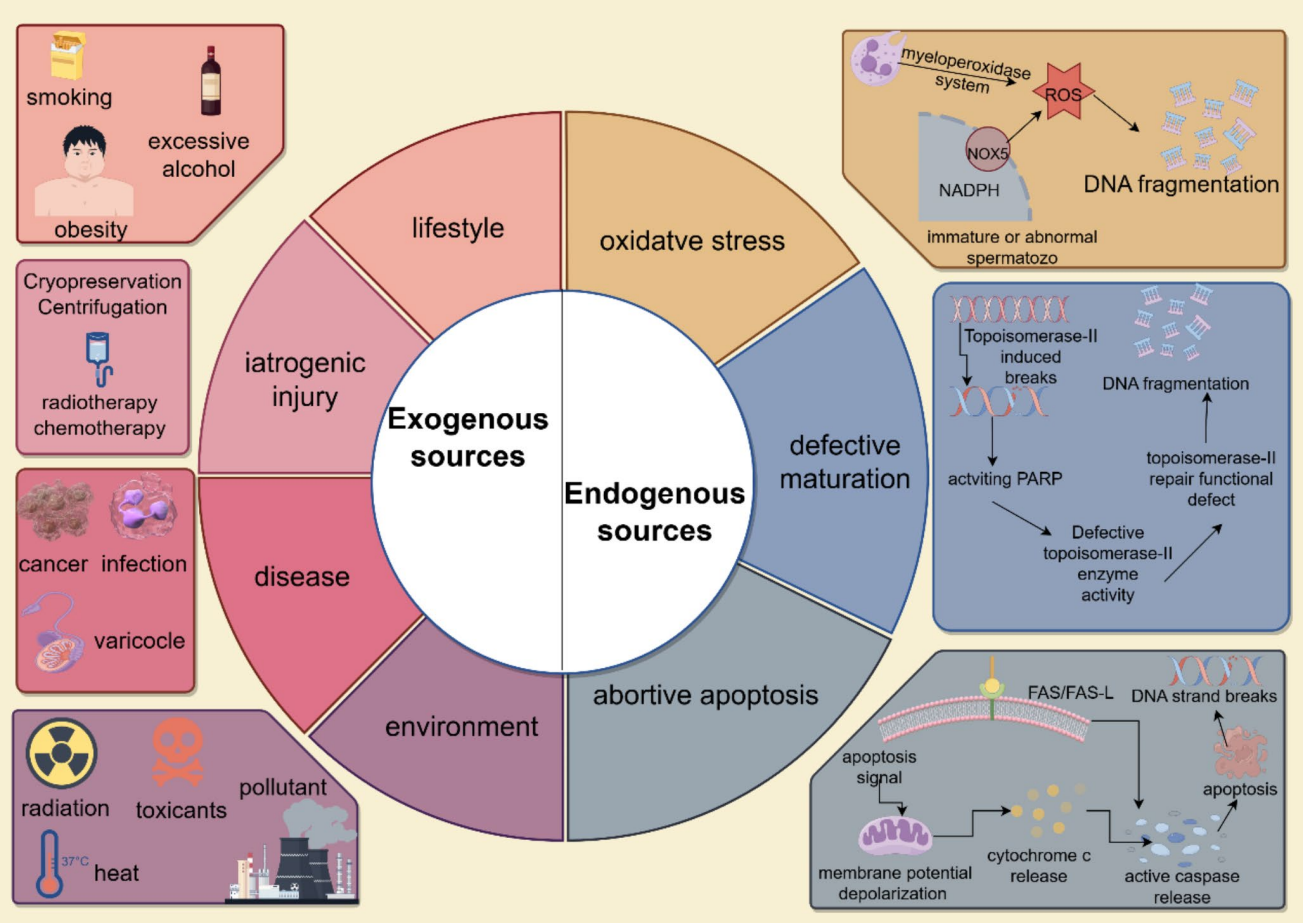
Generalization of factors causing sperm DNA fragmentation. Oxidative stress, sperm defective maturation, and abortive apoptosis are endogenous factors that cause DNA fragmentation. Exogenous factors are poor lifestyle habits (smoking, excessive alcohol, obesity), iatrogenic injury (sperm cryopreservation, centrifugation, radiotherapy, chemotherapy), disease (cancer, infections, varicocele), and the environment (radiation, toxicants, environmental pollutants, heat)

The endogenous factors include oxidative stress (OS), defective maturation, and abortive apoptosis. In case of an infection or inflammation, leukocytes ingest pathogens and activate the peroxidase system, leading to the breakdown of these pathogens and subsequent reactive oxygen species (ROS) production. Moreover, morphologically malformed immature spermatozoa also produce large amounts of NADPH, which in turn generates ROS through the action of NADPH oxidase 5 (NOX5) [28, 29]. When ROS are produced excessively, they can directly cause double-strand DNA breaks and induce apoptosis, or provoke depolarization of the mitochondrial membrane potential (Fig. 1). This leads to the release of the signaling molecule cytochrome C and activation of caspase, which subsequently activates the polymerase via hydrolysis, leading to apoptosis. The Fas/Fas-L system is involved in the apoptosis of spermatogenic cells [30, 31]. There exists an endogenous nuclease, topoisomerase II, during spermatogenesis, that induces single and double-stranded DNA breaks and has an important role in DNA strand breaks and reattachment to establish the condensed state of mature spermatozoa [32]. Whereas DNA strand breaks activate poly(ADP-ribose) polymerase (PARP) and inhibit topoisomerase II, break incisions force DNA to twist, but if not repaired, sperm DNA fragmentation (SDF) may occur in spermatozoa (Fig. 1) [33–35]. The exogenous factors are lifestyle, environment, iatrogenic injury, and disease. Unhealthy lifestyles such as smoking, alcoholism, and obesity can all lead to DNA damage. Exposure to certain chemicals, including phthalates and organophosphate insecticides, and air pollutants such as nitrogen dioxide and sulfur dioxide increases DNA damage [36, 37]. Centrifugation of spermatozoa, sperm freezing and thawing can cause DNA damage during assisted human reproduction techniques. Cancer treatments like radiotherapy and chemotherapy, and certain diseases such as cancer, infections, and varicocele significantly increase DFI [38–40]. Overheating can increase scrotal temperature, causing irreversible damage to spermatogenesis and producing excess ROS that damage DNA. Prolonged exposure to ionizing radiation increases the risk of fragmentation [41].

### Mechanisms of Sperm DNA Damage Repair

**Fig. 2:**
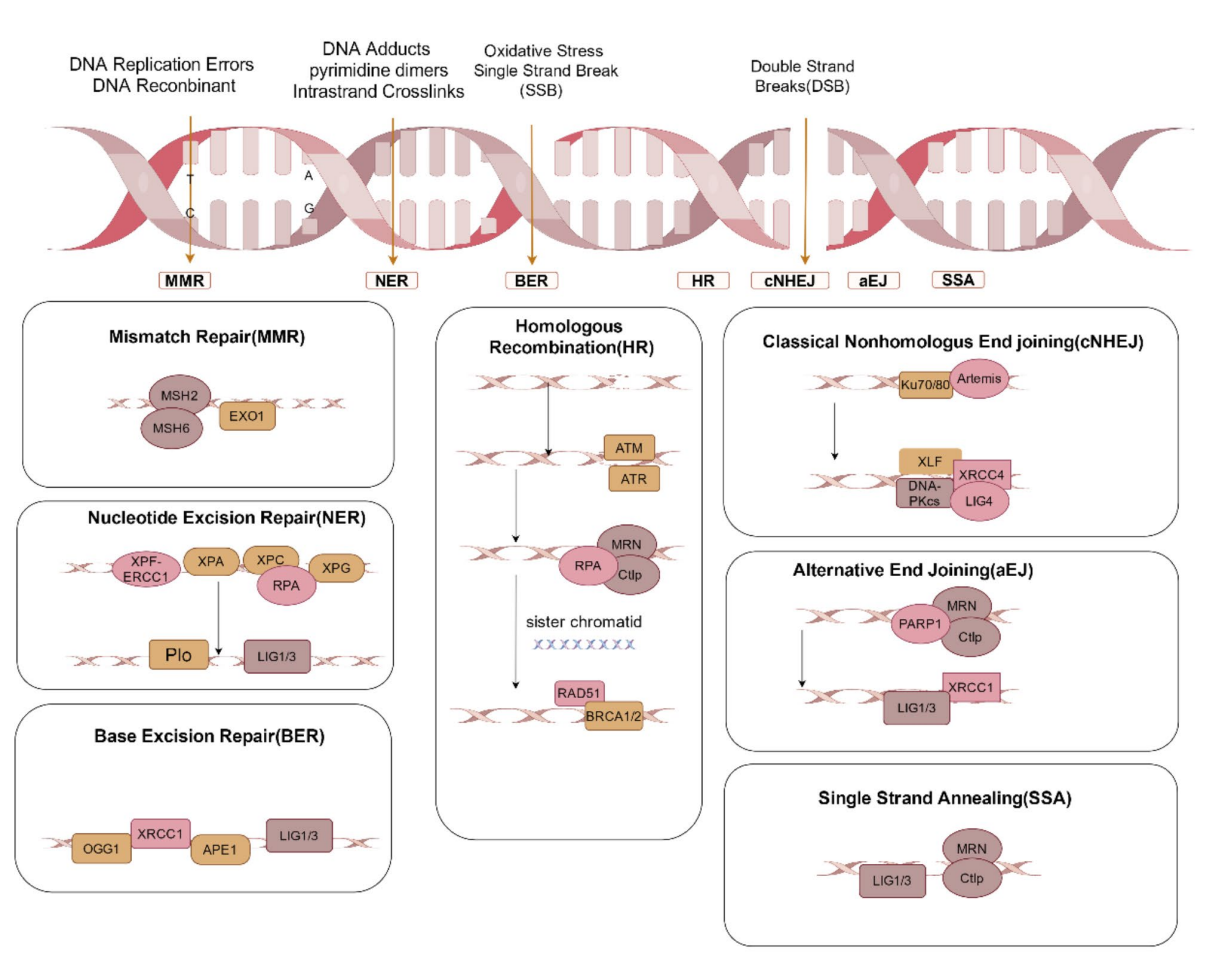
DNA damage repair mechanism. The primary repair mechanisms are NER, BER, MMR, HR, cNHEJ, aEJ, and SSA. APE1: Apurinic-Apyrimidinic Endonuclease I, ATM: Ataxia telangiectasia mutated, ATR: Ataxia rad-related, BRCA1: Breast Cancer protein 1, BRCA2: Breast Cancer protein 2, CtIP: CtBP interacting protein, DNA-PKcs: DNA-dependent protein kinase catalytic subunit, OGG1:8-oxoguanine DNA glycosylase-1, LIG: DNA ligase, MRN: Mre11-Rad50-Nbs1complex, MSH2: MutS homolog 2, MSH6: MutS homolog 6, PARP1: Poly(ADP-ribose) polymerase 1, RAD51: DNA damage repair protein 51, RPA: Replication protein A, XRCC1: X-ray repair cross-complementing protein 1, XRCC4: X-ray repair cross-complementing protein 4, XPA: Xeroderma Pigmentosum group A, XPC: Xeroderma Pigmentosum group C, XPG: Xeroderma Pigmentosum group G, XLF: XRCC4-like factor

The primary repair mechanisms operative in mammalian germ cells are as follows: NER, BER, MMR, and DSBR (Fig. 2) [42, 43].

### DNA Damage Repair Mechanisms during Spermatogenesis

Spermatogenesis, the process of sperm cell production, is divided into three primary stages. Mitotic amplification divisions: Spermatogonia differentiates into primary spermatocytes through a series of mitotic amplification divisions [44]. Meiotic reorganization: The spermatogonia goes through meiotic reorganization to produce haploid spermatocytes. Spermiogenesis: In this final stage, cytoskeletal structures are rearranged to transform round spermatocytes into mature spermatozoa [45]. The process commences with the substitution of histones by transition proteins TP1 and TP2, which are subsequently replaced by protamine P1 and P2 [46]. It’s important to note that irregularly high histone levels in sperm can lead to reduced fertility and an elevated risk of failure post-fertilization.

Spermatogonia cells often encounter endogenous and exogenous attacks during spermatogenesis, which can lead to a variety of DNA damage. To cope with this, these cells are equipped with several defense mechanisms which include different DNA damage repair pathways (Fig. 2), including MMR, NER, BER, and DSBR [47]. DNA damage resulting from nucleotide mismatches is primarily restored by the MMR pathway, with recognition of the damage done by MSH2/MSH6 specifically targeting the region of nucleotide mismatch in the daughter strand. The DNA exonucleases 1 (EXO1) and then remove the damaged portion [48–50]. Mitotic proliferation is a process that occurs during the reproductive lifespan of an organism, resulting in many sperm being produced. The mismatch that occurs during this step is repaired through the MMR pathway. The NER pathway is crucial for eliminating large DNA adducts during spermatogenesis and involves xeroderma pigmentosum protein (XP). It is frequently used in fixing small localized damages and preventing large amounts of endogenous DNA adducts, involving the endonucleases XPA, XPC, XPG, and excision repair cross complementation group 1 (ERCC1), which cut the damaged strand. POL repairs the damage and connects it using DNA ligase 1 (LIG1) [7]. The BER pathway primarily focuses on repairing oxidative base damage through chromatin remodeling. This complex process includes the excision of damaged bases by 11 different DNA glycosylases, resulting in a base-free site for POLβ to bind and complete repair. This is accomplished by forming a complement complex with X-ray repair cross-complementing protein 1(XRCC1), and binding to either LIG1 or DNA ligase 3 (LIG3) [51, 52]. The BER pathway in human spermatozoa is truncated but fully functional, containing only the 8-oxoguanine DNA glycosylase-1 (OGG1) protein. This pathway requires the participation of apurinic-apyrimidinic endonuclease 1 (APE1) and XRCC1 from the oocyte to complete repair after fertilization [53]. A traditional Chinese medicine (TCM) formula, Yishen Tongluo (YSTL), has been demonstrated to repair sperm DNA damage induced by tripterygium glycosides (TGs) in rats by stimulating the BER pathway [43]. KEGG pathway analysis of 119 differentially expressed genes and 158 differentially expressed proteins identified using trend analysis revealed significant enrichment in the BER pathway at the transcriptome level [43].

DSB is the most severe type of damage and needs to be repaired swiftly to maintain male germ cells’ genomic integrity. There are four pathways for DSB repair (Fig. 2), including HR, cNHEJ, aEJ, and SSA [54–56]. HR and cNHEJ, appear to share responsibilities depending on the cell cycle phase and type of DNA breaks. Specifically, HR acts mainly in S-phase and on replication-caused single-stranded DSB to repair damage, whereas cNHEJ acts mainly in G1-phase and on more erroneous neighboring double-stranded DSB [57, 58]. In vertebrates, the majority of DSB are repaired by the cNHEJ pathway. HR pathway is involved in spermatogonia and spermatocytes, cNHEJ pathway is involved in spermatogonia, spermatocytes, round spermatocytes, and spermatids, and aEJ pathway is involved during spermatogenesis from spermatogonia to spermatids ( excluding spermatocytes) [59]. In the HR repair pathway, DSB is first recognized by ataxia-telangiectasia mutated (ATM) or ATM- and Rad3-Related (ATR) and recruits MRE11, RAD50, and NBS1 to form Mre11-Rad50-Nbs1complex (MRN) complex, MRN shears the terminal DNA to form single-strand DNA (ssDNA), ssDNA is encapsulated by replication protein A (RP-A) thus preventing it from being degraded by nuclease, and then through a series of complex regulation, the terminal complex is covered by RP-A and replaced by DNA damage repair protein 51 (RAD51) to form nucleoprotein filament. After a series of complex regulations, RP-A is covered by RAD51 and replaced by RAD51 to form nucleoprotein filaments. Breast cancer protein 1 (BRCA1) or breast cancer protein 2 (BRCA2) binds to RAD51. RAD51 protein enters the DNA double-stranded template and pairs with homologous DNA sequences to form the D-Loop structure, which extends or joins with another end to complete the repair process [59, 60]. Sphingosine-1-phosphate (S1P) has been demonstrated to mitigate DNA damage in spermatozoa by stimulating the ATM-mediated HR pathway. Furthermore, S1P has been shown to alleviate genotoxic stress-induced sperm DNA damage and to enhance the expression of a crucial DNA repair gene, BRCA1 [61].In the cNHEJ pathway, it is mainly formed by Ku70 and Ku80 to form a heterodimer, which attracts the recombinase Artemis, and then convenes DNA-dependent protein kinase catalytic subunit (DNA-PKcs), X-ray repair cross-complementing protein 4 (XRCC4), DNA ligase 4 (LIG4), and XLF to form a complex, which ultimately completes the repair process, in phases G1 and G2 [62]. cNHEJ pathway allows two broken DNA strands to rejoin regardless of sequence homology, making it more error-prone and susceptible to mutation [63]. When the cNHEJ pathway is dysfunctional, the aEJ pathway is triggered. The aEJ pathway is recruited at DNA breaks by the MRN complex for poly(ADP-ribose) polymerase 1 (PARP1), and the ligation is completed by XRCC1 and LIG 1/3 [64, 65]. similarly, the SSA pathway repairs containing over 20 bp of non-conserved homology. Like aEJ repair, it also depends on the cooperation of the CtBP interacting protein (CtIP) and MRN complexes for extensive excision [66]. Interestingly, the capacity to repair DNA diminishes as men age [61].

### Fertilized Oocyte Repair

The maturation of sperm has a limited capacity for DNA repair. Therefore, the oocyte takes on the responsibility of repairing and rebuilding both the female and male genomes [67]. The detailed repair mechanisms are shown in Fig. 2. The type and extent of DNA damage may impact embryonic development. Oocytes possess the BER pathway, and SSB and base-free sites resulting from incomplete SSB repair in mature spermatozoa can be readily repaired. Oocytes effectively repair low levels of SSB, whereas most DSB exceeds the repair capacity of oocytes. DSB DNA damage is at a higher risk of being incorrectly repaired than SSB, leading to deleterious mutations and infertility [68]. While DNA damage can be repaired, fertilization of oocytes by spermatozoa with extensive DSB results in virtually irreparable damage [69]. A negative correlation has been found between high degrees of sperm DNA damage and reproductive success, suggesting that once sperm DNA damage reaches a certain level, it may not be fully repaired [23]. Post-fertilization, the BER pathway functions in the oocyte, where oxidative DNA damage initiates the activation of BER. This process eliminates the oxidized residue 8-OHdG via the enzyme OGG1 [70]. XRCC1, found mainly in rough lineage spermatocytes and round spermatids, acts as a crucial scaffold protein within the BER pathway. It integrates different DNA repair factors to restore ROS-induced, single-stranded DNA breaks [71–73]. XRCC1 participates in various DNA repair pathways, including BER, NER, and aEJ [71]. The OGG1 protein is present in human sperm, but the subsequent repair proteins of the BER pathway, APE1, and XRCC1, are undetectable, and thus the BER pathway is not completed in sperm. Fortunately, oocytes are rich in APE1 and XRCC1 [53, 74]. They assist in sperm DNA damage repair between the S phase of the first embryonic cleavage and division following sperm-oocyte fusion [75, 76]. This short time window, from fertilization to the onset of the S phase, is a highly active period for the oocyte. It needs not only to remove the 8-OHdG residues and repair deletion base sites but also perform additional nuclear remodeling to repair damage to the parental genome. This includes single and double-stranded breaks as well as oxidative attacks on 5-methylcytosine [77].

Oocytes are found to be notably proficient in repairing DNA damage of paternal origin. This proficiency is evidenced by the observed activation of various pathways in mice oocytes such as BER, MMR, NER, cNHEJ, and HR [78]. While somatic cells increase the production of DNA repair enzymes and proteins to repair damaged DNA, mature oocytes are transcriptionally silent and depend on an endogenous supply of pre-synthesized proteins and mRNA transcripts accumulated during folliculogenesis to trigger DNA repair [79]. Oocytes are capable of repairing damaged DNA and maintaining the integrity of the genome from the primordial follicle through to the metaphase II (MII) stage. MII oocytes are highly DDR-competent, exhibiting high levels of expression of genes related to NER, HR, BER, MMR, and cNHEJ in mouse, monkey, and human MII oocytes [50, 80]. The relative expression level of XPC was found to be significantly elevated in MII oocytes in comparison to earlier stages. Furthermore, there was a tendency towards increased relative expression of MSH2 and RAD51, which may be associated with the accumulation of messenger RNA for DNA repair genes during IVM in preparation for fertilization [81]. A proteomic comparison of mouse MII oocytes revealed the presence of 53 proteins involved in DNA damage- and DNA repair-related processes. In contrast, a proteomic comparison of primate oocytes revealed the presence of approximately 37 proteins [82]. Better quality oocytes have greater DNA repair capacity, allowing them to cope with damage to sperm DNA. A positive correlation was revealed between the richness of DNA damage repair proteins and oocyte quality in a pig model [83]. However, the DNA repair capacity of the oocyte begins to decline as the oocyte ages. This reduction is attributed to the decreased expression of critical DNA repair genes including BRCA1, RAD51, and ATM [84–86]. This may result in a reduction in the effectiveness of DNA repair and hurt embryonic development, particularly in the presence of DNA damage. Moreover, obesity might have an impact on the repair process. Recent studies have evaluated that reduced oocyte DNA repair activity could contribute to the low early developmental potential of embryos from overweight patients during in vitro fertilization cycles [87]. Other unfavorable conditions, such as polycystic ovary syndrome (PCOS), also affect egg quality and the ability of sperm to repair DNA damage [88]. In scenarios involving oxidative sperm DNA damage, there exists a risk of promoting a subversion mutation (GC to TA). This risk is heightened if the sperm is not completely repaired by the fertilized oocyte by the first S phase. If the level of sperm DNA damage is below 8%, the oocyte is capable of repairing the damage. However, if the DNA repair of the parental genome by the fertilized oocyte is incomplete, it may result in an increased risk of introducing de novo mutations into the fertilized egg [89]. Although oocytes can repair some DNA damage, cellular apoptosis can occur if the level of DNA damage exceeds the oocyte’s ability to repair the damage fully. Consequently, this could result in reduced embryo quality or even halt embryo development [90, 91].

Increasing oocyte DNA repair capacity may improve sperm DNA repair efficiency, but there is only limited evidence to support this. One clear fact is that oocyte repair efficiency decreases with increasing maternal age.

### Embryonic Repair of DNA Damage

DNA damage that is not repaired during the oocyte stage can be carried forward to the embryonic stage where it may then be repaired by the early embryo. The first two cell divisions within an embryo are primarily governed by maternal genes. Paternal influence begins to manifest from the 4-cell stage. It has also been observed that the destructive effects of sperm DNA damage become increasingly apparent at later stages of embryonic development [91]. Sperm DNA damage can still fertilize oocytes, if there’s DNA damage in fertilized eggs or oogonia of 2-cell stage embryos, it triggers the activation of DNA repair mechanisms. It has been demonstrated that sperm DNA damage results in elevated p53 expression and reduced RAD51 expression at the two-cell stage in an oocyte or fertilized egg. RAD51— a DNA repair enzyme, is consistently distributed throughout the cytoplasm and nucleus in oocytes and fertilized eggs. When DNA-damaging agents or replication inhibitors are present, RAD51 is recruited to the nucleus by BRCA2 [92]. The major DNA repair proteins of the BER and NER pathways (LIG3, OGG1) and two key DNA damage checkpoint proteins (TP53, RAD1) are expressed in fish embryos, suggesting an embryonic response to spermatozoa DNA damage [93]. In response to DNA damage, somatic cells typically undergo the G1/S and G2/M cell cycle checkpoints. In comparison to somatic cells, fertilised eggs undergo novel S-phase checkpoints, enabling the activation of relevant DDR and DNA repair pathways [67]. Damage to the spermatozoon impairs active DNA demethylation of the paternal genome in the fertilized egg, resulting in altered epigenetic reprogramming during early development. DNA fragmentation has implications for later stages of development, and oxidative DNA damage drives the BER to repair DNA, recruiting XRCC1 to the damaged paternal prokaryotic nucleus [94]. Embryonic genome activation (EGA) occurs between the mouse embryo’s two-cell stage and the human embryo’s eight-cell and mulberry stages [95]. Most DNA repair in damaged embryos is probably done before EGA. Following EGA, the embryo assumes greater responsibility for repairing DNA damage. If the oocyte DNA repair mechanisms are insufficient to cope with the damage before EGA, the embryo will need to activate additional repair mechanisms before it can develop and implant, or it will miscarry.

Spermatogenesis, post-fertilization Oocyte, and embryo period can all undergo sperm DNA repair, as summarized in Table 1.

**Table 1:** The mechanisms for repairing sperm DNA damage in spermatogenesis, fertilized oocytes, and embryos

Period	Repair Mechanisms	Components	References
Spermatogenesis	MMR	MSH2/MSH6, EXO1	[48–50]
	NER	XPA, XPC, XPG, ERCC1, POL	[7]
	BER	XRCC1, LIG1, OGG1, LIG3, POLβ	[51, 52]
	HR	ATM, ATR, MRN, RAD51	[59, 60]
	cNHEJ	Ku70, Ku80, Artemis, XRCC4, DNA-PKCs, LIG4, XLF	[61]
	aEJ	MRN, PARP1, XRCC1, LIG1/LIG3	[63, 64]
Fertilized Oocyte	BER, MMR, NER, cNHEJ, and HR	OGG1, XRCC1, AEP1, ATM	[70, 71, 78]
Embryos	HR, BER, NER	RAD51, BRCA2, OGG1, APE1, XRCC1	[92–94]

### Key Repair-Related Proteins

Several repair-related proteins have been identified as playing crucial roles in chromatin condensation, regulation of transcription, repair of DNA damage, and regulation of the cell cycle. As research progresses, several promising proteins are becoming known, and they are being identified as new targets for research in other areas of medicine. Consequently, they are promising targets for male DNA repair studies, as summarized in Table 2.

**Table 2:** Promising repair-related proteins

Protein	Repair Mechanism	Parameters	Expression
XLF PAXX	cNHEJ cNHEJ	XRCC4, DNA-PKcs KU, LIG4, XRCC4, DNA-PKcs	Testis Testis
BRUCE	HR	ATR, ATM	Testis
WIP1	-	γ-H2AX	Placenta, Testis
FASN	cNHEJ	NF-κB pathway, SP1, PARP1	Fat, Testis
ACO2	cNHEJ	NF-κB pathway	Heart, Testis

XLF: XRCC4-like factor, PAXX: Paralog of XRCC4 and XLF, HP1β: heterochromatin protein 1β, WIP1: Wild-type p53-induced Phosphatase 1, FASN: fatty acid synthase, ACO2: mitochondrial aconitate hydratase

### XLF

XLF stimulates LIG4 activity, promotes LIG4 adenylation, and assists in DSB end alignment through the formation of XRCC4/XLF filaments [96]. The cNHEJ pathway is a process that requires synaptic, lysosomal, and polymerase-dependent end processing and joining of DNA ends. XLF-mediated dimerization promotes the processing of populated ends for cNHEJ repair, which is essential for final joining. Critical for final ligation, the XRCC4/XLF complex can bridge DNA in vitro and promote autophosphorylation of DNA-PKcs. It suggests that DNA ends are simultaneously bridged by synaptic DNA-PKs and XRCC4/XLF, which promotes DNA-PKcs autophosphorylation [97]. The inhibition of cNHEJ activity was observed in both cell-free and live-cell assays in knockout XLF cells, accompanied by a high degree of unrepaired cellular DSB. These findings suggest that XLF serves to facilitate DNA end-joining, thereby promoting cNHEJ activity in cancer cells [98]. In the context of cancer therapy, expression of the XLF protein is induced following drug treatment, a process that facilitates the repair of DNA damage [98]. Mice lacking the enzyme XLF exhibit deficiencies in cNHEJ, DNA repair, and brain development [99]. The majority of current research on XLF is focused on its role in tumor and nerve biology. XLF plays an essential role in DNA repair, yet there is no clear evidence that it plays an essential role in repairing DNA damage in sperm. Nevertheless, with the ongoing advancement of XLF research, it may emerge as a promising candidate for future investigation as a repair protein.

### PAXX

PAXX is a more recently identified cNHEJ accessory protein. PAXX and XLF are involved in cNHEJ, and the two proteins exhibit structural similarities. However, they operate in different ways. XLF can promote mismatch and non-adhesive end joining in vitro, whereas PAXX is not. PAXX can by itself promote flat-end joining in a KU-LIG4/XRCC4-dependent manner to promote flat end joining [100]. PAXX serves as a scaffold, facilitating the stabilization of the KU70/80 heterodimer at the DSB site and the assembly and stabilization of the cNHEJ machinery. In the presence of XLF, PAXX facilitates the attachment of non-adhesive DNA ends [101]. Both PAXX and XLF are capable of binding to Ku, with PAXX serving to bridge the Ku80-mediated DNA-PK dimer, while XLF remains structurally intact within the XLF-mediated DNA-PK dimer [102]. It can be observed that PAXX partially compensates for XLF in terms of end-bridging. Furthermore, it can be demonstrated that XLF loss results in a reduction in DNA-PKcs S2056 phosphorylation, as would be expected. Nevertheless, the simultaneous loss of PAXX and XLF exacerbates the defective S2056 phosphorylation caused by XLF loss alone [102]. A study has demonstrated that embryos deficient in both XLF and PAXX genes exhibit a high rate of mortality, which may be attributed to the accumulation of DNA repair defects [103]. chemoresistance to doxorubicin or cisplatin leads to increased PAXX-Ku70 binding and increased cNHEJ efficiency. However, the small molecule M11 disrupts PAXX-Ku70 binding and resensitizes chemoresistant osteosarcoma to doxorubicin and cisplatin. Thus, it suggests that PAXX may represent a promising novel target for overcoming chemoresistance in OS therapy, and PAXX deficiency has been observed to re-sensitize chemoresistant osteosarcoma cells to doxorubicin and cisplatin [104]. PAXX is a relatively novel repair protein that is not yet fully understood. However, it is believed that it will prove to be a promising target for future studies of DNA repair.

### HP1β

Heterochromatin protein 1β (HP1β), encoded by the Cbx1 gene, is actively involved in processes like chromatin condensation, transcriptional regulation, and DNA damage repair [105]. The histone variant H2AX gets phosphorylated by ATM kinase at its C-terminal serine 139 in response to DSB, leading it to be known as γ-H2AX. It is a crucial DNA damage marker [106]. γ-H2AX helps collect more ATM to increase its levels and recruit other DDR proteins. Functionally, deletion of HP1β has been linked to increased expression of γ-H2AX, defects in DNA replication, and associated DNA damage that affects spermatogenesis [107].

### BRUCE

BRUCE is a DNA damage response protein that aids in the stimulation of ATM and ATR for the HR pathway in cells [108]. In a mouse study where BRUCE was systemically knocked out, spermatocytes often showed persistent DNA breaks. Lack of ATM and ATR signaling in non-synaptic regions of rearranged autosomes implies an HR impairment of ATM- and ATR-dependent DNA damage responses in meiosis. This reveals the critical role of BRUCE in DNA damage repair [109].

### WIP1

Wild-type p53-induced phosphatase (WIP1) has long been recognized as playing an important role in autophagy. WIPI proteins are responsible for the detection of this pool of newly produced PI3P, acting as indispensable PI3P effector proteins that facilitate the recruitment of downstream autophagy-related proteins [110]. WIP1 plays a significant role in restoring the cell to its pre-stress state by deactivating DDR signaling activity via protein phosphatase after DSB repair is complete. Inhibition of WIP1 during fertilization reduces γ-H2AX expression significantly in both paternal and maternal prophages, suggesting its role in improving the repair of fertilized eggs [25]. Despite a dearth of research on its role in DNA repair, the protein in question is nevertheless worthy of further study.

### FASN, ACO2

Differential expression of the proteins fatty acid synthase (FASN) and mitochondrial ouabain hydratase (ACO2) was found to be involved in DNA damage repair in varicocele semen samples. Increased expression of FASN on the one hand inhibits the NF-κB pathway, ACO2, and oxidative stress. On the other hand, it can induce overexpression of specificity protein 1 (SP1), which increases the expression of PARP1 and enhances the cNHEJ pathway [111, 112]. A reduction in ACO2 expression subsequently results in DNA damage [111].

### Summary and Prospects

The influence of males on reproductive outcomes is just as crucial as that of females. Integration of damaged sperm DNA into the embryonic genome can lead to errors in DNA replication, transcription, and translation during embryonic development, leading to human disease or fetal miscarriage. Before conceiving a child, the male should undergo DFI testing. This ensures a healthy pregnancy by reducing miscarriage risk and preventing human diseases caused by DNA abnormalities. A range of interventions, such as adjusting lifestyle habits, abstaining from ejaculation, taking oral antioxidants, employing hormone therapy, repairing varicoceles, and retrieving sperm from the testicles have been applied to tackle the factors leading to sperm DNA damage. These measures aim to enhance sperm quality and reduce DFI [90, 113, 114]. Moreover, gene therapy could be a potential treatment for DNA damage caused by the deletion of specific genes. The repair of sperm DNA relies primarily on spermatogenesis and post-fertilization by oocyte or embryo development. However, research on the mechanism of repair after fertilization is limited. Several important proteins are involved in the repair mechanism. The repair-related protein XLF and PAXX are involved in the cNHEJ pathway and are essential for the final ligation [97, 101]. The function of XLF and PAXX in repairing DNA damage in sperm has not been reported, making it a potential new area of research in sperm DNA damage repair.

## Data Availability

Not applicable.
